# Motivation for alcohol consumption or abstinence during pregnancy: A clinical-qualitative study in Brazil

**DOI:** 10.1371/journal.pone.0223351

**Published:** 2019-10-04

**Authors:** Júlia Lustosa Martinelli, Carla Maria Ramos Germano, Lucimar Retto da Silva de Avó, Bruno José Barcellos Fontanella, Débora Gusmão Melo

**Affiliations:** Department of Medicine, Federal University of São Carlos, São Paulo, Brazil; Bielefeld University, GERMANY

## Abstract

**Background:**

In Brazil, alcohol consumption is estimated to range from 7 to 40% in pregnant women. This research investigated the motivation for alcohol consumption or abstinence during pregnancy in a purposive sample of Brazilian women.

**Methods:**

Clinical-qualitative research was conducted, in which 14 women participated, identified as risk-drinkers during pregnancy by the T-ACE screening tool. Data were collected through semi-structured individual interviews. Reports were recorded, transcribed and investigated by a thematic content analysis approach. The results were discussed based on a comprehensive and interpretive framework.

**Results:**

Sixteen themes were extracted and these were then classified into three thematic categories: (a) general motives for alcohol use, (b) specific motives for drinking during pregnancy, and (c) reasons for partly or fully abstaining from drinking during pregnancy. Social motives were the most relevant, particularly due to deeply rooted cultural values. Enhancement motives were widely mentioned and associated with a hedonic posture. Consumption also aimed to deal with negative emotions, characterizing two types of coping specifically to pregnancy: first, perceiving pregnancy as a period of less opportunity for social interaction and, therefore, drinking alcohol at home to compensate; secondly, perceiving pregnancy as a period of greater irritability, and hence experiencing difficulties to abstain. On the other hand, concern about fetal health, disapproval of consumption by relatives and health professionals, as well as the social construction of mothering were reported as reasons to abstain.

**Conclusions:**

Some specificities influence the decision to continue or discontinue alcohol consumption during pregnancy. To reduce consumption, we suggest educational actions based on a collective health perspective, articulated with individualized measures that allow identification and proper intervention for women at risk.

## Introduction

At present, alcohol is the most common teratogenic agent in the world [1]. Research in Canada, the US and Europe indicate that the frequency of drinking during pregnancy ranges from 10 to 50%, depending on the instrument used to identify consumption [2–5]. In Brazil, alcohol consumption is estimated to range from 7 to 30% in pregnant women [6–9]. In a study conducted by our group in 2015, in the city of São Carlos, São Paulo, Brazil, 7.3% of women were identified by the T-ACE screening tool as risk-drinkers during pregnancy [10].

The current state of evidence and medical recommendations are to avoid any prenatal alcohol consumption [2, 11–13]. In Brazil, the latest guidelines published by the Ministry of Health recommend that primary healthcare professionals should advise pregnant women about the risks and advocate abstinence, especially in the first three gestational months [14]. In 2017, the Brazilian Society of Pediatrics launched a campaign called “Pregnancy without alcohol,” in which it recommends total abstinence in all gestation periods [15]. Despite this, mild to moderate consumption by pregnant women seems to be generally accepted by Brazilian society [16].

The literature has sought to identify factors that comprise a complex set of cognitions and behaviors that culminate in alcohol consumption [17]. In a general context, dispositional constructs, such as the expectations about and the motives for consumption are considered of great clinical importance [18]. The motives for drinking alcohol are considered the final and common way through which more distal factors, such as personality traits and positive expectations, are related to the use of this substance [19, 20]. The motives indicate to what extent individuals use alcohol to achieve certain internal or external goals, and can be defined in terms of needs or functions fulfilled by alcohol use [19]. Thus, motives can only be evaluated in individuals who use alcohol, since they are cognitive-affective elements that help maintain use once it has started; an individual may, thereby, use alcohol for more than one motive at the same time [20]. Identifying information about specific functions that alcohol has in individuals can contribute to developing more effective preventive strategies [21, 22]. These dispositional constructs, although consolidated into the general population, have been underestimated and not always applied as tools to comprehend alcohol intake during pregnancy.

One of the main theoretical models concerning the motivations to use alcohol was hypothesized by Cooper (1994) [21] based on the conceptualization proposed by Cox and Klinger (1988) [19]. It is called the “four-factor model” and proposes the existence of four distinct motives that influence the individual decision: (1) social motives, e.g., drinking to liven up parties or celebrate with others; (2) enhancement motives, e.g., drinking to maintain or amplify positive affect; (3) coping motives, e.g., drinking to avoid or dull negative affect; and (4) conformity motives, e.g., drinking to avoid social disapproval by a group [19, 21, 23]. Previous studies correlated the different motives to patterns of consumption and personality traits. Particularly in women, it has been suggested that coping motives are associated with neuroticism and anxiety [24–26]. Enhancement, social and coping motives were related to quantity and frequency of alcohol use [18]. Conformity motives, on the other hand, showed a poor correlation with these indicators [18] and were related to the use of alcohol in specific situations to avoid social rejection [20]. No previous studies were identified in our literature review in which the “four-factor model” was directly applied to pregnant women. This is, therefore, a pioneering approach to the theme.

While motives for alcohol consumption play a prominent role in the cognitive models that explain the decision to drink [21, 23], the complementary construct of motivation to abstinence has been much less studied [27, 28]. A consolidated model for the motivation for alcohol abstinence among pregnant women was also not identified in the literature review. As well as the motivation for consumption, the motivation for abstinence may be related to certain expectations and drinking patterns, possibly helping to prevent and treat alcohol use disorders [29].

Although there is a vast body of literature on prevalence, associated factors and potential harm from to pregnant women by alcohol consumption, studies of how women themselves interpret alcohol use during pregnancy are scarce. Qualitative research in this sense began in the last decade and is believed to be important in issues such as prevention and distribution of information, as well as the approach of these women and health professionals by whom they are oriented [30, 31]. Research conducted on the subject has shown that social and enhancement motives, as well as intimacy with people who consume alcohol, plays a prominent role in consumption maintenance [16, 32]. On the other hand, the health of a developing baby was described as the main motive for habit change and abstinence [6, 33].

We did not identify explanatory or interpretive models for alcohol use during pregnancy contextualized for the Brazilian scenario in the literature. The characterization of the motivation for alcohol consumption and the reasons for abstinence can be useful for clinical interventions, as well as for planning public health education policies. This justifies the relevance of this subject from a scientific and social point of view, which prompted the development of the present study.

## Methods

### Setting

The study was carried out in São Carlos, a city located in the State of São Paulo in Southeast Brazil. There are approximately 230,000 inhabitants and the geometric growth rate is approximately 1% per year. In 2010, the human development index was 0.805, despite the considerable Brazilian social inequality in this municipality. In São Carlos, access to prenatal care is higher than the average in the State of São Paulo: in 2014, for example, 86.39% of pregnant women had seven or more prenatal consultations, while in the State 76.69% of pregnant women reached this number. Most prenatal care is performed in the Brazilian Unified Health System (called SUS in Portuguese, *Sistema Único de Saúde*), which is universal and free for everyone [34].

The research was approved by the Human Research Ethics Committee at the Federal University of São Carlos (process 1.540.308), and all participants provided written informed consent (S1 File).

### Study design and sampling plan

This is an exploratory study, developed using clinical-qualitative methodology [35]. The group of participants, defined purposively, consisted of 14 adult women, who were pregnant during the second half of 2015 and the first half of 2016. These women participated in a previous study developed by our group [10] and were identified as risk-drinkers during pregnancy by the Brazilian validated revised T-ACE screening tool [13, 36]. The T-ACE screening tool is a questionnaire consisting of four main questions; a score is assigned for each one of these questions and the maximum value of the questionnaire is equal to 5. In the Brazilian version, the four main questions of the T-ACE are interspersed with nine other questions that address habits during pregnancy (sleep, eating, etc.) and do not interfere with the outcome of the instrument. These interspersed questions are used to make the women feel relaxed during the interview so that they do not trigger their defense mechanisms, assuming a position of denial and omitting fundamental information that could make it impossible to identify alcohol consumption [13]. When answering the questionnaire, whoever reaches a score of ≥ 2 is considered as a risk-drinker, i.e., as an alcohol consumer that is potentially sufficient to damage the fetus [7, 36]. Each T-ACE question takes about one minute to ask and it represents the first validated sensitive screen for risk-drinking appropriate for routine use in obstetric-gynecologic practice [36].

In total, 59 T-ACE positive women were selected as possible participants. Women were contacted by telephone and invited to participate in the research, referred to primarily as a study about pregnancy habits and alcohol consumption to which they were invited to due to alcohol consumption identification in our previous research [10]. Not all women answered the phone call; therefore, researchers were only able to contact 21 out of the 59 women by the telephone numbers indicated in our previous study. We consider that this difficulty in reaching the women can be explained by the high rates of renting houses and temporary residence in the studied population. Among the women reached, five refused to participate saying they were too busy or overloaded at the time. Interviews were then conducted with 16 women, considering two acculturation interviews and 14 interviews effectively included in the *corpus* to be analyzed. Therefore, the selection process of participants may be defined as purposive and convenience sampling [37, 38].

### Characteristics of participants

Table 1 presents a sociodemographic characterization and the alcohol ingestion patterns of the participants.

**Table 1:** Participants’ sociodemographic characterization.

Characteristics	N (%)
**Age range (years)**	
20–25	4 (28.5)
> 25–30	6 (42.8)
> 30–35	3 (21.4)
> 35	1 (7.1)
**Skin color (self-declared)**	
Black	6 (42.8)
White	5 (35.7)
Mixed race	3 (21.4)
**Education level**	
Elementary school graduate	3 (21.4)
High school graduate	9 (64.3)
University graduate	2 (14.3)
**Relationship status**	
Married/stable relationship	14 (100)
**Monthly familiar income**	
Between 1 and 2 minimum wages	5 (35.7)
Between 2 and 3 minimum wages	3 (21.4)
Between 4 and 5 minimum wages	2 (14.3)
> 5 minimum wages	4 (28.5)
**Religion**	
Catholic	10 (71.4)
Protestant Christian religions	3 (21.4)
African Brazilian religion (Umbanda)	1 (7.1)
**Prenatal care setting**	
Brazilian National Health System	9 (64.3)
Supplementary health system	5 (35.7)
**T-ACE score**	
T-ACE = 2	11 (78.6)
T-ACE = 3	1 (7.1)
T -ACE = 4	2 (14.3)
**Alcohol consumption pattern**	
Sporadic (related to events)	2 (14.3)
Once a month	1 (7.1)
Twice a month	2 (14.3)
Weekly (during weekends)	8 (57.1)
Three times a week	1 (7.1)
**Alcohol consumption pattern during pregnancy**	
^a^Complete abstinence after recognizing pregnancy	3 (21.4)
Abstinence after the seventh month of pregnancy	1 (7.1)
Partial reduction after recognizing pregnancy	2 (14.3)
Maintenance of previous consumption pattern	7 (50)
Initiation of alcohol consumption during pregnancy	1 (7.1)

^a^Participants 4, 12 and 13.

### Data collection

The sociodemographic profile of the participants was investigated using a pre-defined questionnaire (S2 File) including information on age, skin color, schooling, religion, marital status, number of children, family income and work.

Data was collected by conducting individual in-depth semi-structured interviews [39] from September 2016 to August 2017. To improve the reliability of the data collected, we used some validation strategies. Only one female interviewer conducted the face-to-face interviews. She was considered skilled only after two acculturation talks with two women, in which she attempted to familiarize herself with their general problems and the interview script. The participants were not the interviewer’s patients; therefore, they could express some content without being afraid of clinical judgments. A comfortable setting for the interviews (participants’ homes) was guaranteed; the university was also offered as an option for the interview, however all participants chose their own residence as a preferable setting. A relationship of trust between the interviewer and interviewees was encouraged as the written informed consent form was explained in detail to them and anonymity was ensured. The participants’ validation was not used, as the main research topic was very sensitive.

The trigger question was: Why do you drink alcoholic beverages? Based on this issue, the participants’ personal reflection and free expression were encouraged. During the interviews, some key themes in a pre-defined script (Table 2 and S3 File) were proposed. Open-ended questions were developed based on the researchers’ clinical experience, considering the study purposes and exploratory nature of the research. The questions were adapted to the lexicon of the participants and presented openly, allowing more depth in the expression of personal meanings to the women’s responses.

**Table 2:** Semi-structured script used in individual interviews.

*Trigger question*: *Why do you drink alcoholic beverages*?
1. Can you tell me more about your alcohol consumption? How did it start?
2. What do you think about alcohol consumption?
3. How do you feel when you drink alcohol?
4. What do you expect to happen when you drink alcohol?
5. Do you think that drinking helps or hinders you in any aspects?
6. In which kind of situation do you feel more like drinking? In which do you feel inhibited?
7. How do you believe approval or disapproval from people surrounding you influenced your alcohol consumption?
8. Have there been any changes in your attitude to alcohol during pregnancy?
9. How do you believe alcohol interferes in your daily life? And in the baby’s intrauterine development?
10. Do you believe pregnancy is a period of less or more fun?

The interviews were recorded (ranging from 30 to 50 minutes) and transcribed. The auditory and written records comprised the *corpus* of the research.

### Data analysis

The thematic content analysis approach, as proposed by Bardin in 1977, was adopted to examine the *corpus* [40]. Operationally, all the significant statements in the *corpus* were open coded (that is, we did not use pre-set codes), and then some clusters of codes were identified as themes [41]. Then, these themes were circumscribed into thematic categories, forming analogical groups, classified and aggregated according to what was understood about their meanings for the study participants [40, 41].

The interviews were analyzed and coded one by one separately by three researchers, who had carefully read the *corpus*. The convergent and divergent aspects of the individual analyses were discussed in groups, and the consensual themes and thematic categories presented in the results were developed progressively. This analysis led to a theoretical saturation of the results, as we had sufficient elements to reach the proposed objectives, and we verified that new themes did not appear in significant numbers in the last interviews [42, 43].

Meeting the aims of the research and based on frequency of appearance and clinical relevance (even if a theme was identified in only one or two interviews), we selected the main themes in the three thematic categories considered. The category “general motives for alcohol use” was defined as a priori and it was based on a theoretical reference on motivation for alcohol consumption [19, 21], while the categories “specific motives for drinking during pregnancy” and “reasons for partly or fully abstaining from drinking during pregnancy” were more inductive and established a posteriori during the interview analysis. Although this is qualitative research, we opted to quantify certain issues spontaneously mentioned (Table 3), illustrating what seemed to be more relevant to the participants in relation to the proposed questions. Regarding the category called “specific motives for drinking during pregnancy,” the number of times the themes were mentioned is relatively small, possibly reflecting participants’ greater cultural or psychological difficulties to verbalize such kinds of contents. Even so, we defined them as “themes,” because we considered these verbalizations to be clinically very significant.

**Table 3:** Main themes in the different thematic categories and their frequencies.

Thematic categories		Frequency^a^	
**General motives for alcohol use**		**Number of interviews**	**Total**
**Themes**	Social motives.	12	56
	Enhancing motives.	12	34
	Coping motives.	7	23
	Alcohol taste related motives.	6	12
	Seeing other people drink makes the alcohol user want to drink.	4	6
	Conformity motives.	3	10
	Alcohol intake takes place due to habit, no specific motive identified.	3	7
**Specific motives for drinking during pregnancy**		**Number of interviews**	**Total**
**Themes**	Drinking is a way of reassuring independence and, during pregnancy, prohibition (not being able/permitted to drink) can be an additional motive for wanting to drink–desire increases when prohibited.	3	13
	Cravings for alcohol (craving is considered “uncontrollable” and must be satisfied).	3	12
	Specific pregnancy coping: increased irritability/sadness during the period, consuming alcohol to lighten a negative mood.	2	3
	Specific pregnancy coping: less opportunities of social interaction and fun during pregnancy, consuming alcohol at home to make up for it.	1	3
**Reasons for partly or fully abstaining from drinking during pregnancy**		**Number of interviews**	**Total**
**Themes**	Partial or total abstinence after recognizing pregnancy due to concern about fetal health, associated with fear/guilty feelings.	6	33
	Partial or total abstinence after pregnancy or around children is justified due to maternal responsibility (“maternal role”), unlike the father, who is not subjected to this responsibility (sexist point of view).	7	22
	Believes reaffirmation of risks associated to alcohol consumption during pregnancy by the healthcare professional would have a positive impact on stopping consumption.	7	10
	Family/friends disapproval influences abstinence during pregnancy.	4	7
	Partial or total abstinence and development of other “healthy” activities during pregnancy are supported by spirituality (God gives strength, courage, thank God for stopping drinking/smoking, etc.).	4	4

^a^The number of participants who mentioned the theme can be seen in the column “number of interviews”. The number of times the theme appeared throughout all the 14 interviews is presented in the “total” column.

Finally, the thematic categories and respective themes were examined and discussed using the literature background and the clinical experience of the authors, aiming to adopt a comprehensive and interpretive approach of the results.

## Results

The distribution of the 16 themes selected in three distinct thematic categories can be seen in Table 3.

### General motives for alcohol use

Social motives were the most frequent reasons for alcohol use from participants, seeming to be due to deeply rooted cultural and familiar values. The secondary socialization process that some participants had experienced also seemed to have been strongly intermediated by alcohol consumption, most of the time beginning in adolescence, promoting consumption naturalization and possibly making it difficult to change the habit during pregnancy. Women highlighted pleasure and opportunities for socialization, similar to the importance in maintaining affective bonds through in-group alcohol consumption and identification with partners. During the interviews, participants expressed the thought that freedom, socialization environments and situations would favor consumption. Some participants reported a desire to drink being aroused by seeing others drink, which would be possibly classified as a social motive, but also comprehensible as a conformity issue.

“I think I feel more like drinking when I’m at a party. I think it’s because of the vibe, the music, the environment, the people.” (Participant 8)

“If someone says, ‘Let’s go and have a drink,’ I say ‘OK, let’s go, let’s go together.’ I value more this thing of being together. If not, then I don’t drink.” (Participant 11)

“Personal interaction. Not drinking is just like going to a party where you stay outside and everyone else goes in.” (Participant 14)

“Sometimes you just invite someone round for lunch and they bring a crate of beer. So, you already get drunk.” (Participant 5)

“If you go somewhere where people aren’t drinking, why would you drink in front of everyone? You’re not going to drink. So yes, it has some influence.” (Participant 14)

Enhancing motives were also widely mentioned, usually associated with a hedonic posture of looking for pleasure, fun, relaxation, and a better mood to do household chores and take care of the children.

“I’m already a bubbly person, so I get even more bubblier. I talk more, laugh a lot, talk a lot. I need a drink to clean up the house, to get it done quicker.” (Participant 6)

“I am happier, so I talk more, speak more. I can have a bit of fun.” (Participant 9)

“In a certain way it’s pleasurable. So, I said, ‘I won’t give it up while I’m pregnant.’” (Participant 11)

Some women still associated consumption to coping with negative emotions, thus taking advantage of some of the psychotropic effects of alcohol and characterizing, therefore, coping motives.

“Like, I’m sad, I’m down, I’m gonna drink. Of course, it won’t solve anything, but it’s as if I was drowning my sorrows.” (Participant 1)

“I drink to relax, to calm down. ‘Cause two children aren’t easy. They get on my nerves!” (Participant 6)

“Sometimes I’m so stressed out with everyday life. Then comes that pain, that depression, so I go and get a beer.” (Participant 9)

Conformity motives, among the motives of the “four-factor model” [19, 21], were the least relevant for the women who were interviewed. Consumption was also referred to as a routine/pattern, without any specific motive identified. The taste for alcohol, mainly wine and beer, was also expressed.

“I think when you start hanging out with people who drink, sometimes you wanna try it and end up liking it, right?” (Participant 5)

“There are always people at my home, so someone always goes out to buy beer. So, I see them drinking and I have a glass of beer.” (Participant 5)

“I have no reasons for drinking. I drink because I’m used to it, actually. You always do it, so you want it.” (Participant 7)

“I think it was more because of the taste. Wine is really good. I really like it.” (Participant 4)

### Specific motives for drinking during pregnancy

Dealing with the pregnancy period itself was described as a drinking motive. Two specific coping motives were identified: first, the perception of pregnancy as a period for less opportunity of social interaction and fun, with compensatory alcohol consumption at home. Second, the perception of pregnancy as a period of greater sadness/irritability and discontent with their body image, with the idea that abstinence would make the period more painful.

“It was the way I found to distract myself and have fun, even though I was at home. But I’m drinking, having fun, listening to music. I didn’t go out, but I’m drinking. As I couldn’t go, I’m gonna drink. It helps me have some fun, having a bit of distraction.” (Participant 1)

“I thought, ‘I’m not going to go out like that, with my belly this size, nothing fits me.’ So, I guess I just stopped having some fun.” (Participant 1)

“I would have gotten more stressed out if I hadn’t drunk during pregnancy. It would have been harder.” (Participant 6)

Another specific motive to drink during pregnancy was related to cravings for alcohol. Cravings for alcohol were understood as solid barriers to abstinence, considering the difficulty women faced controlling the will for consumption. There was even a perception that alcohol consumption due to craving would be forgiven/legitimated, because of its “uncontrollable” character (Participant 3). This motivation was expressed specifically in one of the interviews, in which the participant, a religious practitioner, did not consume alcohol before pregnancy. Another two women also mentioned this aspect.

“I can’t explain it. It’s uncontrollable. Some women have these crazy cravings. People eat earth, soap, whatever. I went for drinking, which I found pretty strange.” (Participant 3)

“You know, women during pregnancy crave stuff. I craved wine. A lot.” (Participant 4)

“We might think it [craving] is silly until we get pregnant, and then find out it’s true. One day when I was pregnant, I craved beer. I said, ‘I need to have a beer, my mouth is watering.’” (Participant 8)

Some women expressed their motivation to drink as a way of coping with social prohibitions (from family members or partners), apparently aiming at reaffirming their independence.

“I’m pretty stubborn. If I hear someone saying, ‘Going to drink, are you?!’, then I’ll do it just out of stubbornness. Just to make it clear that no one bosses me around.” (Participant 9)

This theme was also understood as a coping strategy towards the social judgment of drinking during pregnancy, by showing indifference towards criticism.

“I would even drink something, and me pregnant, some people would look, others wouldn’t. So, I said, ‘I don’t care. I didn’t really care about what people were thinking.’” (Participant 9)

One of the participants also mentioned that the recommendation to abstain/ban consumption during pregnancy made abstinence more difficult, increasing the desire for alcohol and becoming a specific drinking motive.

“You know, just one sip while cooking lunch, even on my own. So, then I downloaded a pregnancy app on my phone and it said ‘You must not consume alcoholic beverages.’ God, it seemed I was in the Sahara Desert. I strangely craved for beer. You crave it three times more.” (Participant 14)

### Reasons for partly or fully abstaining from drinking during pregnancy

Although most women consider pregnancy as a period marked by psychological conflicts between consumption and abstinence, some participants reported partial or even total abstinence after recognizing pregnancy. Among the motives, concerns about the fetus and newborn growth and development were frequently cited.

“I feared that she would be born with some body part missing. Besides being retarded. Because we know how the world is nowadays. People reject others, they don’t really care, ain’t that right? So, I thought, ‘how is that girl going to grow up with some body part missing?’ Arriving at kindergarten and being laughed at by her friends.” (Participant 4)

“I kept imagining if I drink, he would too. People say, ‘the baby eats everything we eat.’ So, I thought what if he wiggles around, and rolls himself up in the [umbilical] cord.” (Participant 12)

Although generally substantiated in empirical popular basis (a kind of spontaneous epidemiology), some beliefs were remotely connected with scientific knowledge, while others exclusively had a religious background (“If God believes it will happen, then it will; everything happens through God’s permission.”–Participant 6). Commonly the damage to the baby was referred to as proportional to the amount of alcohol intake in each drinking episode (“A glass or two, I don’t think it’s bad; three would be too much.”–Participant 5). From this point of view, there seems to be a spontaneous posture of damage control: one participant, for example, conveyed her “moderation” saying she would only drink beer, not liquor, only during the weekends and only at home (Participant 6). Another woman justified abstinence because of the specific fear of abortion, due to a personal history of losses. The perception of “everything or nothing” was also presented: abortion would happen, or pregnancy would follow without any other negative outcomes. Fear of other negative outcomes apart from abortion, such as malformation and intellectual disability, as well as the feeling of guilt, were also recurrent. Sometimes, having previous knowledge or experiencing negative outcomes in another women’s pregnancy was relevant.

“I was pregnant when I heard about the negative outcome. Because she [a friend] said her nose was all flat, and the doctor said it was because of all the alcohol her mom consumed. So, I saw the whole thing, and I said wow, so this is no lie, right? So, I cut back a little.” (Participant 11)

“I would even try it, but I was scared something would happen to the baby.” (Participant 13)

“I drank up to 5 months. Then I found out about the pregnancy, and I stopped. And, you see, I even felt like it, but I said, ‘I can’t, because of my daughter.’ Because I thought about her health.” (Participant 4)

“You don’t know if it’s gonna be really bad for the baby. So, you end up cutting back a bit.” (Participant 10)

Consumption disapproval by family, friends or healthcare professionals was also referred to as a reason to abstain. Reassurance, from healthcare givers, about the risks associated with alcohol consumption during pregnancy would have, in some women’s opinion, a positive impact on stopping drinking. On the other hand, it was supposed that, sometimes, healthcare professionals were only aiming to intimidate or frighten women, not being a reliable source. The lack of consistent information was perceived as a reason for keeping the pattern.

“Sometimes it seems they [healthcare professionals] are only saying it to scare you.” (Participant 6)

“I won’t drink because of them, not even secretly. Because if she [mother] finds out, oh God!” (Participant 7)

“If she [the doctor] came to me and said ‘You must not drink anything,’ I wouldn’t. I wouldn’t risk my child’s life because of a beer, I wouldn’t drink. But when she [doctor] said it was ok, I sort of got laid-back.” (Participant 14)

“We just hear it’s not good, but what can it cause? I don’t know. So, I’d like to have more information. And that’s also a medical mistake. I think the doctor should inform us, mainly mothers who are having their first baby. I think if I’d had this information since the beginning, I would definitely have done it differently. But since I had the information that a glass of wine per day was fine, I said ‘Why not?’ The doctor knows what he is talking about.” (Participant 11)

Some gender issues were mentioned by the participants: abstaining to not expose their behavior to their children (as opposed to the father, who would not have this “responsibility”); and the perception of the drinking habit as something “ugly” for the woman, but not for the man.

“When my daughter is around, I avoid it. Her dad drinks more. I’m the mum, so I always have to be sober.” (Participant 4)

“Drinking is already disgusting for a man, imagine for a woman.” (Participant 2)

“It’s the mum’s child, not the dad’s. It’s the mum’s responsibility. That’s why the mum can’t drink. Imagine a drunk mum, the child keeps waking up all night long and she can’t manage to get up.” (Participant 14)

## Discussion

To the best of our knowledge, this is the first study to make a qualitative interpretation of drinking during pregnancy through the “four-factor model” [19, 21]. As emphasized by Meurk et al. (2014) [30], despite numerous quantitative studies on alcohol consumption, there are surprisingly few qualitative studies presenting the perspective of women who choose to drink during pregnancy, particularly on its emotional dimension. This is an important knowledge gap whose filling has great potential for improving public health policies and discussions between practitioners and patients regarding alcohol use during pregnancy and should, therefore, be analyzed in more depth [30]. Among the motives to drink that are described in the “four-factor model” [19, 21], the participants of our research tended to refer, overall, to social and enhancing motives followed by coping motives; conformity motives were the least mentioned throughout the interviews.

In agreement with previous studies [30, 32, 44, 45], imposed cultural alcohol consumption in social events was widely cited as a reason to make exceptions for abstinence. These occasions were seen as moments when women missed drinking freely, in the wake of the old tradition of associating alcohol to leisure and socialization [46]. These data are also consistent with another study carried out with 40 Brazilian pregnant women in which all women who had a habit of consuming alcoholic beverages during pregnancy did it in the presence of friends, family and partners, corroborating the recreational character associated with alcohol consumption [16]. The role of alcohol in reinforcing marital, family and friendship bonds was also expressed in our research. Specifically, consumption to strengthen the bond with the partner became apparent (“I like to have a beer with my husband, because we chat, we catch up, it’s really nice, you know!”–Participant 11), corroborating with data from Esper [47] and reinforcing the importance of including the partner in interventions to decrease consumption, which can result in greater effectiveness [48]. Accordingly, recent research conducted in Sweden, where antenatal care comprises an appointment in week 6–7 for counseling about lifestyle issues, including alcohol, showed that most partners decreased their alcohol consumption in the expectation of parenthood, claiming a sense of responsibility for the pregnant partner [49]. Considering these results, a broader approach, focusing on women’s social context and, most specifically, on partners, seems indispensable for them to have a significant role in women’s abstinence. We suggest conducting public awareness campaigns about the effects of alcohol consumption during pregnancy, through printed and audiovisual educational materials.

Coping motives were also widely cited by our subjects, corroborating with another qualitative study in which Crawford-Williams et al. [33] observed that women felt more stressed during pregnancy because of money, the partner or the pregnancy itself, and they believed that the stress could be more detrimental to the fetal health than alcohol exposure [33]. In addition to the motives described in the literature, dealing with the childbearing period itself emerged as a specific motivation. Although pregnancy, birth and maternity are culturally associated to positive emotions, it is known that this period can be stressful for some women [50]. Some of the participants expressed the perception of pregnancy as a period of less opportunity for social interaction and leisure time, as well as a period of greater sadness, irritability and dissatisfaction towards their own body, which would make abstinence even harder. Another study performed in South Africa found similar results: alcohol consumption as a strategy to deal with stress or negative emotions, including those associated with pregnancy and to maintain social connections through pregnancy [51]. The childbearing period brings along different specificities that directly or indirectly influence the decision about maintenance or cessation of the drinking habit. It can be assumed that in developing countries, such as Brazil and South Africa, there is less social support for women to deal with the gestational period than in developed countries, making the phenomenon even more complex.

One of the participants reported that the recommendation to abstain during pregnancy made abstinence more difficult, increasing alcohol desire. Drinking as a way of confronting social sanctions (from family or partners) and of reassuring independence was also expressed. Transgression of tradition and pre-established order are considered attitudes towards obtaining autonomy and individuality, and alcohol is commonly used in this context, for example, by teenagers [52]. This representation also agrees with the research developed by Garcia [53] among Brazilian groups of Alcoholics Anonymous. In this research, the participants considered that, because of the social construction of the feminine gender role, consuming alcohol in public spaces is a transgression, leading to the discriminative categorization of “women who drink”.

Our study also showed that, in general, the emphatic abstinence recommendation during pregnancy was not accompanied by consistent information about the potential harm of consumption to the fetus. There seems to have been, in fact, a clash between an excessive amount of divergent, unspecific recommendations, coming from different sources, and a lack of dependable information available through reliable sources, such as healthcare professionals. This is also consistent with international data, in which women reported that the information that their healthcare practitioners provided regarding alcohol use and pregnancy were limited and superficial [54]. In another study, women described their healthcare providers as being “relaxed” about the risk of alcohol consumption [30].

Although drinking motives have a prominent role in cognitive models for explaining alcohol consumption, the complementary construct of abstaining motives has been largely neglected [30, 31]. Our data point out that concern about the newborn baby’s health and fear of negative nonspecific outcomes were the principal reasons for some interviewed women’s partial or total abstinence. Other qualitative and quantitative research also corroborates with this data, showing that the developing baby’s health was the main motivation for abstinence and this decision was, most of the time, guided by fear or guilt of possible unknown or uncertain harms, instead of an informed and careful resolution [6, 30, 31, 33]. Information and advice given by people close to them were also expressed as a motivation for abstinence in our study, corroborating with a British study in which women reported great influence from family and friends in this decision [55]. Once again, we highlight the importance of public health actions aimed not only at women but also to their social context.

A variety of complaints was expressed by the subjects about healthcare professionals’ clinical approach and the Brazilian healthcare system. In spite of this, the finding that an emphatic orientation from the healthcare professionals about risks associated with alcohol consumption would have, for some subjects, a positive impact on habit cessation, reinforces the importance of a clear, didactic and broad approach to risks in prenatal settings. Healthcare professionals must be updated on the subject and realize their potential to manage pregnant women’s concerns [31]. Brazilian research showed that 22.7% of the physicians who work in prenatal settings do not know about the potential harm of alcohol intake during pregnancy, and some of them even recommend up to a glass of wine, now and then, to their patients [56]. Another Brazilian study developed with pregnant women who drank during the childbearing period showed that only 43% were advised about abstinence [57]. Thus, it is essential to invest in training health care professionals, so more uniform orientations can be given aiming at total abstinence throughout the whole pregnancy, transmitting clear and objective information, as well as establishing intervention protocols in appropriate cases.

The importance of addressing the subject during prenatal care was highlighted by Jones et al. [58], who concluded that to effectively deal with alcohol consumption during pregnancy, it is essential to have an appropriate approach toward alcohol intake as a regular component of prenatal care. It is important to emphasize that for over twenty years, the American College of Obstetricians and Gynecologists and the American Academy of Pediatrics have advised US clinicians to question current and prior alcohol use at the first prenatal visit for all pregnant women. There is also a warning about how alcohol use during pregnancy is stigmatized and how denial is common, reinforcing the importance of the routine utilization of well-validated and reliable methods to detect it, such as the T-ACE questionnaire [58]. Once a woman is identified as an alcohol user, the employment of techniques of Brief Intervention, involving counseling through motivational interviews conducted even by a non-specialist in substance use disorders, may be sufficient for reducing or eliminating the risks associated with maternal exposure to alcohol for most women [12, 48, 59, 60].

The participants in our research also emphasized the idea of abstinence associated with maternal responsibility, as opposed to the paternal responsibility, which does not imply in abstinence. These results corroborate with a study carried out by Jones and Telenta [32], in which women showed a conflict between social pressure for alcohol consumption and the demand to fulfill the image of “good mother”, and can be understood considering the social construction of motherhood. In a sociological perspective, Badinter [61] believes that maternal love, as it is known nowadays, emerged at the end of XVIII century, and, being a value both natural and social, favorable to the species and society, this promoted a radical change in the image and role of motherhood [62]. Thus, the care of women with their children would not be restricted to fulfilling basic needs, but it would also demand a psychic availability aiming to reach the adequate emotional development of the child, which would depend on the effectiveness of the maternal care [63]. According to Rubin [64], the author who introduced the concepts of “maternal identity” and “maternal role attainment” in the 1960s, pregnancy is a preparation period for the woman to become a mother in a psychosocial context. Nowadays, a widespread western model of motherhood has been defined as “intensive mothering”: an ideology that defends extremely demanding motherhood practices in terms of energy, expenditure, money, and efforts [65]. Thus, the relation between alcohol and females can also be understood as a gender issue, in which familiar bonds, moral conceptions, advertisement models and social classes are articulated considering consumption patterns [66], indicating that interventions must be made not reinforcing gender role stereotypes and considering the specificities of female gender and motherhood social constructions.

### Strengths and limitations

Despite the research constraints in terms of the external validity, since the sample of participants was formed by convenience, we consider that internal validity of the results was significant, as the employed data collection technique provided ample opportunities for free expression to participants, thus enabling an initial approach of the subject in the Brazilian context.

A key limitation of the study is that the T-ACE survey is unable to distinguish between alcohol occasional use, moderate use, and heavy use. Even though the current medical recommendation is to avoid any prenatal alcohol consumption, the general population are unaware of the effects of moderate and occasional prenatal use of alcohol. Motives to drink or abstain should be based on these norms and, thus, one would expect motives for heavy drinking to differ from those for moderate drinking. Additionally, motivations to decrease or to stop prenatal alcohol use were analyzed together. A further limitation of our study includes the fact that the interviews were conducted within two years of the pregnancy and this can be a long time for an accurate retrospective recall of drinking motives.

### Implications for policy and practice

The results enable us to propose some issues to be considered by health authorities in Brazil. Current Brazilian ministerial recommendations for primary healthcare should be revised in agreement with international recommendations, and therefore the subject of alcohol consumption should be systematically approached during prenatal care. This could be done by screening pregnant women using validated instruments, such as the T-ACE questionnaire, as well as by more emphatic recommendations about alcohol abstinence during pregnancy by health professionals.

We reinforce the importance of public campaigns on the harmful effects of alcohol use in pregnancy aimed not only to women but also to their social context. We also consider the need to inform people about the risk of alcohol use in pregnancy, in any quantity and at any time, on alcoholic beverage packaging.

## Conclusions

This qualitative study provides in-depth information to better understand the motivations for alcohol consumption or abstinence during pregnancy in a purposive sample of Brazilian women. Some specificities influence the decision to continue or discontinue alcohol consumption during pregnancy. To reduce alcohol consumption, we suggest instigating educational actions based on public health perspectives, as well as a systematic approach of alcohol intake during prenatal care, articulated with individualized actions, which enables proper and early identification and intervention for women, if necessary.

## Supporting information

S1 File Informed consent form. (DOCX)

S2 File Sociodemographic questionnaire. (DOCX)

S3 File Semi-structured script used in individual interviews in Portuguese. (DOCX)
