# Supplementary material for: Motivation for alcohol consumption or abstinence during pregnancy: A clinical-qualitative study in Brazil
Source: PLoS One. 2019 Oct 4;14(10):e0223351. doi: 10.1371/journal.pone.0223351 (PMC6777787; doi:10.1371/journal.pone.0223351)
Supplement: S1 File — (DOCX) [file pone.0223351.s001.docx]

**Termo de Consentimento Livre e Esclarecido (TCLE)**

**Pesquisadoras**: Estudante Júlia Lustosa Martinelli, Profa. Dra. Carla Maria Ramos Germano, Profa. Dra. Débora Gusmão Melo.

**Instituição**: Universidade Federal de São Carlos, Departamento de Medicina.

**Pesquisa**: Expectativas e motivações acerca do consumo de álcool durante a gravidez: um estudo clínico-qualitativo

Somos da Universidade Federal de São Carlos (UFSCar) e estamos interessadas em entender as expectativas e motivações para o uso de álcool durante a gestação.

Você está sendo convidada para participar dessa pesquisa, intitulada “Expectativas e motivações acerca do uso de álcool durante a gravidez: um estudo clínico-qualitativo”. Sua participação é totalmente voluntária e não haverá qualquer tipo de prejuízo para você ou sua família se você se recusar em colaborar com esse projeto de pesquisa. Entretanto, a sua colaboração é fundamental para que possamos alcançar o resultado pretendido.

Caso você aceite participar da pesquisa, nós gostaríamos de entrevistá-la. Primeiro, preencheremos um questionário com informações sociais e demográficas, como por exemplo: cor de pele, religião, estado civil, escolaridade, profissão e renda familiar. Em seguida conversaremos sobre a sua última gestação, como esteve a sua saúde, as informações e recomendações que você recebeu, a sua visão dos riscos e benefícios de certos hábitos e os motivos que te influenciaram a ingerir álcool ou não durante a gestação. A análise dessas informações nos informará sobre quais orientações estão sendo passadas para as gestantes, e também sobre a maneira como as mulheres recebem essas informações e a percepção que elas têm sobre o uso de álcool durante a gestação.

As entrevistas serão gravadas e devem durar aproximadamente quarenta minutos. Todas as informações que você apresentar serão mantidas em sigilo, jamais serão divulgadas relacionadas ao seu nome ou da sua família e somente serão utilizadas para esse estudo. A estudante fará a transcrição da fala gravada para um texto e as pesquisadoras discutirão esses resultados. Ao término da pesquisa os resultados serão divulgados em encontros científicos e revistas especializadas, contendo citações anônimas, sem que seu nome apareça associado à pesquisa.

O único risco possível nessa pesquisa será o de você ficar angustiada ou desconfortável em conversar sobre algumas coisas relacionadas à sua gestação e aos seus hábitos. Caso isso ocorra, as pesquisadoras se comprometem a esclarecer as dúvidas que possam surgir.

Não há benefícios diretos ou imediatos para você ou sua família por participar desse estudo, além da oportunidade de você poder falar sobre suas percepções, mas esperamos que as conclusões do estudo possam beneficiar gestantes e mulheres que querem engravidar.

Sua participação deverá ser inteiramente voluntária, ou seja, sem qualquer tipo de pressão. Você poderá desistir de participar em qualquer momento da entrevista. Além disso, a sua participação na pesquisa não envolverá qualquer despesa financeira de sua parte. Se você concorda em participar, por favor, preencha os campos abaixo.

Considerando as colocações acima, eu, ________________________________, aceito participar deste estudo e, sendo minha participação totalmente voluntária, estou livre para a qualquer momento desistir de colaborar na entrevista, sem qualquer prejuízo para mim ou minha família.

Eu recebi uma cópia deste Termo e tive a possibilidade de lê-lo.

Assinatura da entrevistada:_______________________________________________

Assinatura da entrevistadora:_______________________________________________

São Carlos, _______/_______/________.

**Endereço e telefone para contato com as pesquisadoras**

**Departamento de Medicina - UFSCar - tel. (16) 3351-8340 ou 3351-8978**

[**juliamartinelli@outlook.com**](mailto:juliamartinelli@outlook.com)**;** [**dgmelo@ufscar.br**](mailto:dgmelo@ufscar.br)

Comitê de Ética em Pesquisa em Seres Humanos da UFSCar

Pró-Reitoria de Pós-Graduação e Pesquisa da UFSCar

Rodovia Washington Luiz, Km. 235 - Caixa Postal 676 - CEP 13.565-905

São Carlos - SP - Brasil.

Fone (16) 3351-8110. Endereço eletrônico: [cephumanos@ufscar.br](mailto:cephumanos@ufscar.br)
