# Supplementary material for: Motivation for alcohol consumption or abstinence during pregnancy: A clinical-qualitative study in Brazil
Source: PLoS One. 2019 Oct 4;14(10):e0223351. doi: 10.1371/journal.pone.0223351 (PMC6777787; doi:10.1371/journal.pone.0223351)
Supplement: S2 File — (DOCX) [file pone.0223351.s002.docx]

**Questionário sociodemográfico**

**Idade da participante**:______________.

**Cor de pele referida do participante** (IBGE)

( ) branca ( ) preta ( ) parda ( ) amarela ( ) indígena

**Cor de pele percebida do participante** (IBGE)

( ) branca ( ) preta ( ) parda ( ) amarela ( ) indígena

**Estado civil**

( ) solteira ( ) casada ( ) relação estável (amasiada) ( ) divorciada

( ) viúva ( ) 2º casamento/relação estável ( ) 3º ou mais casamento/relação estável

**Religião (IBGE):** __________________________________________________________________________.

**Praticante:** ( ) sim ( ) não

**Escolaridade**

( ) ensino fundamental incompleto ( ) ensino fundamental completo (antiga 4ª série ou primário)

( ) ensino médio incompleto ( ) ensino médio completo (antigo 2º grau ou secundário)

( ) formação técnica ( ) ensino superior completo ou incompleto

( ) pós-graduação latu sensu (MBA, curso de especialização, etc.)

( ) pós-graduação stricto sensu (mestrado, doutorado)

**Profissão**:_______________________________

**Número de filhos vivos**:_____________________________.

**Na última gestação (2015 ou 2016), fez pré-natal**: ( ) SUS ( ) Saúde suplementar

**Fez quantas consultas no pré-natal?** ___________________.

**Tabagista? ( ) Sim ( ) Não**

**Quantos cigarro/dia? _______________________________.**

**Renda domiciliar** (renda da família / número de pessoas que moram na casa; salário mínimo = R$880,00)

( ) até ½ salário mínimo ( ) entre ½ e 1 salário mínimo

( ) entre 1 e 2 salários mínimos ( ) entre 2 e 3 salários mínimos

( ) entre 3 e 5 salários mínimos ( ) acima de 5 salários mínimos
