# Supplementary material for: Motivation for alcohol consumption or abstinence during pregnancy: A clinical-qualitative study in Brazil
Source: PLoS One. 2019 Oct 4;14(10):e0223351. doi: 10.1371/journal.pone.0223351 (PMC6777787; doi:10.1371/journal.pone.0223351)
Supplement: S3 File — (DOCX) [file pone.0223351.s003.docx]

**Roteiro semiestruturado para entrevista**

| ***Pergunta disparadora: Por que você consome bebida alcoólica?*** |
| --- |
| 1. Você pode me falar mais sobre o seu consumo de álcool? Como começou? |
| 1. O que você pensa sobre o consumo de álcool? |
| 1. Como você se sente quando bebe álcool? |
| 1. O que você espera que aconteça quando consome álcool? |
| 1. Você acredita que beber te ajuda ou te atrapalha em algo? |
| 1. Em quais situações você sente mais vontade ou mais inibida para beber? |
| 1. Como você acha que a aprovação ou não das pessoas ao seu redor influenciou o seu consumo de álcool? |
| 1. Houve mudanças na sua relação com as bebidas alcoólicas durante a progressão da gestação? |
| 1. Como você acha que o uso de álcool interfere na sua vida? E no bebê em desenvolvimento intraútero? |
| 1. Você acredita que a gestação seja um período de menor ou maior diversão? |
